# Saturated free fatty acids and apoptosis in microvascular mesangial cells: palmitate activates pro-apoptotic signaling involving caspase 9 and mitochondrial release of endonuclease G

**DOI:** 10.1186/1475-2840-4-2

**Published:** 2005-01-10

**Authors:** Rangnath Mishra, Michael S Simonson

**Affiliations:** 1 Division of Nephrology, Department of Medicine, School of Medicine, Case Western Reserve University and University Hospitals of Cleveland, Cleveland, Ohio 44106, USA; 2 Division of Nephrology, Department of Medicine, School of Medicine, Case Western Reserve University and University Hospitals of Cleveland, Cleveland, Ohio 44106, USA

**Keywords:** free fatty acids, microvascular, apoptosis, caspase 9, lipotoxicity, mesangial cells

## Abstract

**Background:**

In type 2 diabetes, free fatty acids (FFA) accumulate in microvascular cells, but the phenotypic consequences of FFA accumulation in the microvasculature are incompletely understood. Here we investigated whether saturated FFA induce apoptosis in human microvascular mesangial cells and analyzed the signaling pathways involved.

**Methods:**

Saturated and unsaturated FFA-albumin complexes were added to cultured human mesangial cells, after which the number of apoptotic cells were quantified and the signal transduction pathways involved were delineated.

**Results:**

The saturated FFA palmitate and stearate were apoptotic unlike equivalent concentrations of the unsaturated FFA oleate and linoleate. Palmitate-induced apoptosis was potentiated by etomoxir, an inhibitor of mitochondrial β-oxidation, but was prevented by an activator of AMP-kinase, which increases fatty acid β-oxidation. Palmitate stimulated an intrinsic pathway of pro-apoptotic signaling as evidenced by increased mitochondrial release of cytochrome-c and activation of caspase 9. A caspase 9-selective inhibitor blocked caspase 3 activation but incompletely blocked apoptosis in response to palmitate, suggesting an additional caspase 9-independent pathway. Palmitate stimulated mitochondrial release of endonuclease G by a caspase 9-independent mechanism, thereby implicating endonuclease G in caspase 9-indpendent regulation of apoptosis by saturated FFA. We also observed that the unsaturated FFA oleate and linoleate prevented palmitate-induced mitochondrial release of both cytochrome-c and endonuclease G, which resulted in complete protection from palmitate-induced apoptosis.

**Conclusions:**

Taken together, these results demonstrate that palmitate stimulates apoptosis by evoking an intrinsic pathway of proapoptotic signaling and identify mitochondrial release of endonuclease G as a key step in proapoptotic signaling by saturated FFA and in the anti-apoptotic actions of unsaturated FFA.

## Background

Recent evidence suggests that intracellular accumulation of saturated free fatty acids (FFA) in vascular cells contributes to lipid-mediated cellular damage (see [1-4] for review). The cellular dysfunction associated with FFA overload, known as lipotoxicity, contributes to cell injury in settings of high FFA or triglycerides, such as obesity or type 2 diabetes [4]. Diverse mechanisms have been proposed to explain lipotoxicity including dysregulation of cell signaling, induction of a proinflammatory and prothrombotic state, or in some cases programmed cell death [1,4]. Indeed, saturated FFA have previously been shown to induce apoptotic cell death that is prevented in most cell types by unsaturated FFA [5-14]. In the microvasculature, the pro-apoptotic signaling pathways induced by saturated FFA and the anti-apoptotic pathways regulated by unsaturated FFA remain incompletely understood.

Several mechanisms have been implicated in apoptotic cell death induced by saturated FFA. Some studies suggest that increased β-oxidation of FFA does not contribute to apoptotic cell death and suggest that unmetabolized FFA might be involved [1,13]. However, other studies contradict this observation and suggest a direct role for mitochondrial β-oxidation in the apoptotic response to palmitate.[10]. The finding that long-chain saturated but not unsaturated FFA cause apoptosis implicates a product made specifically from the saturated species. For instance, saturated but not unsaturated FFA are precursors for the pro-apoptotic lipid ceramide. Although palmitate does increase *de novo *ceramide synthesis in cultured cells, studies of the functional role of ceramide in palmitate-induced apoptosis have yielded conflicting results that might depend on the cell type in question [8,13,14]. Because saturated FFA are poor substrates for cardiolipin biosynthesis, decrements in cardiolipin and increased release of mitochondrial cytochrome-c have recently been implicated in apoptosis in breast cancer cells and cardiomyoctyes exposed to palmitate [12,13]. Another recent study demonstrated mitochondrial release of cytochrome-c in palmitate-treated pancreatic β-cells [14], which suggests that an intrinsic mitochondrial pathway of pro-apoptotic signaling might mediate the effects of saturated FFA on cell death.

In the present study, we investigated the hypothesis that the saturated FFA palmitate induces apoptosis in microvascular mesangial cells and delineated the proapoptotic signals involved. We chose to study mesangial cells because lipids accumulate in mesangial cells *in vivo *in experimental models of type 2 diabetes, obesity, or hyperlipidemia [15-20], but the functional consequences of FFA accumulation are unclear. We report here that palmitate induces an intrinsic proapoptotic signaling pathway in mesangial cells that proceeds by a caspase 9-dependent pathway and by a caspase 9 -independent mechanism involving mitochondrial release of endonuclease G. In addition, we demonstrate that unsaturated FFA block both the caspase 9-dependent and -independent pathways of palmitate-stimulated apoptosis.

## Methods

### Reagents

Antibodies used in these studies were as follows: human-specific active fragment of caspase 9 and human cytochrome-c (Cell Signaling, Beverley MA,), human active fragment caspase-8 and caspase-2 (BD Biosciences), endonuclease G (Chemicon, Temecula, CA), and β-Actin (Sigma, #A5316). Cell-permeable inhibitors of caspase 9 (Z-LEHD-FMK) and caspase-8 (Z-IETD-FMK) were from R&D systems (Minneapolis, MN). Etomoxir and 5-aminoimidazole-4-carboxamide-1-β-D-ribonucleoside (AICAR) were from Sigma and Toronto Research Chemicals (Ontario, Canada), respectively.

### Preparation of FFA-albumin complexes

Fatty acid-albumin solutions were prepared by the protocol of Spector [21]. Briefly, sodium salts of FFA (Nu-Chek Prep, Elysian, MN) were added to PBS and gently warmed to facilitate solubility without damaging the fatty acid [21]. The warm, clear fatty acid salt solution was complexed to 5% fatty acid-free BSA in PBS at a 6:1 fatty acid to BSA molar ratio. The sterile filtered, complexed fatty acid solution was added to the serum-containing cell culture medium to obtain the indicated final FFA concentration. The final FFA concentration in the medium was confirmed with an enzymatic colorimetric assay (NEFA C, Wako). We also confirmed that addition of the complex to culture medium did not significantly alter the pH.

### Apoptotic cell death in cultured human mesangial cells

Human mesangial cells (HMC), purchased from Cambrex Bioscience Inc. (Walkersville, MD), were maintained in Dulbecco's modified essential medium (Gibco-BRL) supplemented with 17% fetal bovine serum (FBS), 100 U/ml penicillin, 100 μg/ml streptomycin, 5 ng/ml selenite, and 5 μg/ml each of insulin and transferrin. Characterization was performed by phase contrast microscopy and by immunostaining for intermediate filaments and surface antigens as described previously [22]. Briefly, cells were positive for desmin, vimentin, and myosin, but did not stain for factor VIII, keratin, or common leukocyte antigen.

To measure endogenous levels of cleaved caspase-3, cells were lysed in a buffer containing 20 mM Tris, pH 7.5, 150 mM NaCl, 1 mM EDTA, 1 mM EGTA, 1 % Triton X-100, 2.5 mM Na pyrophosphate, 1 mM β-glycerophosphate, 1 mM Na_3_VO_4_, and 1 μg/ml luepeptin. After adjusting for cell protein (DC Assay, BioRad, Hercules, CA), the amount of cleaved human caspase-3 (Asp 175) was measured by ELISA (Cell Signaling). For Western blotting of cleaved caspase proteins, the cells were washed with ice cold PBS and scraped in CHAPS extraction buffer(50 mM Pipes/HCI, pH 6.5, 2 mM EDTA, 0.1% Chaps, 20 μg/ml leupeptin, 10 μg/ml pepstatin A, 10 μg/ml aprotinin, 5 mM DTT, 2 mM Na pyrophosphate, 1 mM Na_3_VO_4_, and 1 mM NaF) and centrifuged at 2,000 × g for 10 min at 4°C. Protein content in the supernatant was assayed with the DC protein assay. An aliquot of the lysate (25 μg protein) was boiled in SDS sample buffer, resolved on a 4–12% SDS-PAGE gradient gel, and transferred to a 0.2 μm nitrocellulose membrane. After blocking in 5% non-fat dried milk in TBS-T (20 mM Tris-Cl, pH 7.5, 150 mM NaCl, 0.05% Tween 20) for 1 h, the membrane was washed 3 times with TBS-T for 5 min each and incubated overnight at 4°C with primary antibody in 3% BSA in TBS-T. After incubating with suitable HRP-labeled secondary Ab (1:2,000) and extensive washing, the proteins were detected by chemiluminescence with an average exposure ranging from 10–30 sec. As previously described [23], the Western blots were analyzed by densitometry in NIH Image by normalizing values for the relevant caspase fragment to the highest value within each experiments (maximum level = 1).

To quantify the number of pyknotic nuclei, HMC on coverslips were washed once with PBS and fixed for 20 min with freshly-prepared 3.7% formaldehyde/20% sucrose in PBS. After washing twice with PBS the HMC were stained with 5 μg/ml Hoechst 33342 (Molecular Probes, Eugene OR) and mounted in Slow Fade Light (Molecular Probes). Using a Nikon Diaphot microscope, the number of pyknotic nuclei were counted and expressed as a percentage of the total number of nuclei counted (n = >300 nuclei per condition).

DNA fragmentation was assessed by measuring release of nucleosomal fragments into the cytosol (Cell Death Detection ELISA Plus, Roche). Briefly, HMC in 24-well plates were centrifuged *in situ *for 10 min at 200 × *g *and the supernatant gently removed. The monolayer was incubated in lysis buffer for 30 min at room temperature and centrifuged again at 200 × *g*. The supernatant (i.e., cytosolic fraction) was assayed immediately for nucleosomal fragments.

### Enzymatic assay of caspase 9 in HMC

HMC treated with FFA were lysed (20 mM Tris, pH 7.5, 150 mM NaCl, 1.0% Triton X-100) and frozen at -40°C. Equivalent amounts of total HMC protein were added to buffer containing the LEHD caspase 9 peptide substrate linked to a cleavable luciferase substrate, aminoluciferin (Promega). The amount of light produced in a coupled reaction with luciferase was measured once every hour for 3 hours in a Berthold Luminometer. Experiments with increasing amounts of cell protein confirmed that the assay was in the linear range under the conditions described.

### Measurements of cytochrome -c and endonuclease G redistribution

To analyze cytochrome-c redistribution in HMC treated with FFA, cells were fractionated into cytosol and membrane fractions using 0.05% digitonin in an isotonic sucrose buffer exactly as described by Dong and coworkers [24]. Because cytochrome-c release occurs mostly from mitochondria, Western blot analysis of cytosol and membrane fractions is expected to reflect cytochrome-c translocation from mitochondria to the cytoplasm. In separate experiments, the same protocol was used to assess release into the cytoplasm of endonuclease G.

## Results

### Saturated but not unsaturated FFA cause apoptosis in cultured HMC

**Figure 1:**
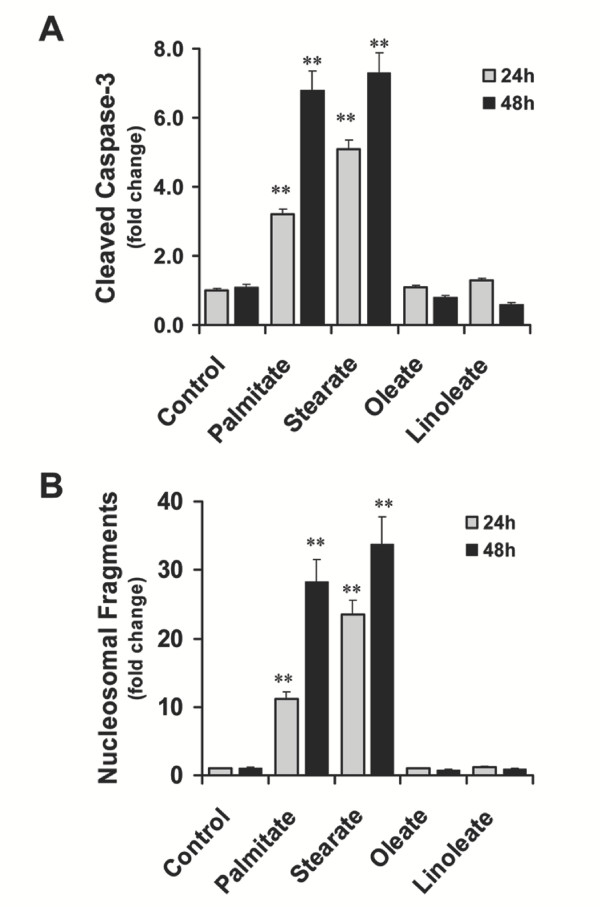
Saturated but not unsaturated FFA induce apoptosis in cultured HMC. HMC were plated for 24 h in media containing 17% FBS, then incubated starting at time 0 with the indicated FFA at 0.2 mM complexed to albumin or with albumin alone (Control), all in media containing 5% FBS. After 24 and 48 h, the level of (A) the cleaved fragment of caspase-3 and (B) cytoplasmic nucleosomal fragments were determined by ELISA and expressed relative to the 24 h control value. Data are mean ± SEM for n = 3 independent experiments in triplicate. **, *P *< 0.01 by ANOVA versus control, oleate, or linoleate alone.

**Figure 2:**
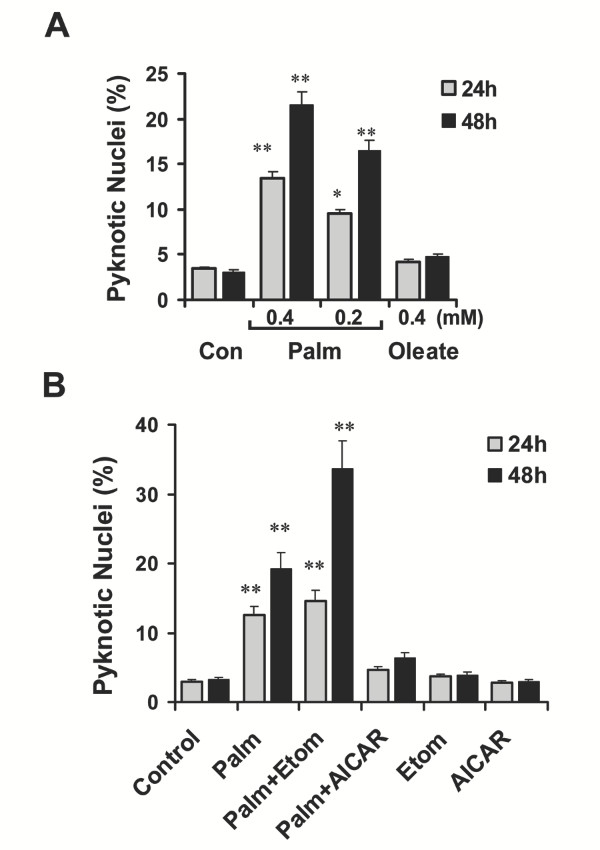
Palmitate-induced formation of pyknotic nuclei and the role of mitochondrial β-oxidation. (A) HMC were treated with palmitate (Palm) or oleate as described in Fig. 1, and the number of pyknotic nuclei were counted after 24 and 48 h in the presence of FFA. (B) HMC incubated with 0.2 mM palmitate in the presence and absence of 0.2 mM etomoxir (Etom) or 0.5 mM AICAR. Cells were also incubated with the same doses of etomoxir or AICAR alone. Data are mean ± SEM for n = 3 independent experiments in duplicate. **, *P *< 0.01 versus control (Con) or oleate alone (A) by Chi-square test.

Intracellular accumulation of long-chain saturated FFA (i.e., palmitate C16:0) has been shown to induce apoptosis in several cell types including cardiac myocytes and pancreatic β-cells [5,6,8,11,13]. To determine whether FFAs induce apoptosis in HMC, cells were incubated in medium supplemented with palmitate, stearate, oleate, or linoleate. The saturated FFA palmitate and stearate increased apoptosis in HMC as evidenced by cleavage of caspase-3 and DNA fragmentation (Fig. 1A,B). In contrast, the unsaturated FFA oleate and linoleate did not increase caspase-3 cleavage or DNA fragmentation compared to cells incubated with albumin alone (Control, Fig. 1A,B). The number of pyknotic nuclei, another prototypical feature of apoptotic cells, were significantly higher in palmitate-treated cells whereas the number of pyknotic nuclei in cell incubated with oleate were similar to control (Fig. 2A). To determine whether mitochondrial β-oxidation was necessary for palmitate-induced apoptosis, the number of pyknotic nuclei were measured in cells treated with etomoxir, an inhibitor of carnitine palmitoyltransferase I. Etomoxir significantly amplified palmitate-induced formation of pyknotic nuclei at 48 h (Fig. 2B). The number of pyknotic nuclei was unaffected by etomoxir alone compared to control. In contrast, stimulating fatty acid oxidation with AICAR, an activator of AMP-kinase, abolished the increase in pyknotic nuclei with palmitate (Fig. 2B). The doses of etomoxir and AICAR used here have been previously demonstrated to inhibit and stimulate, respectively, fatty acid β-oxidation [13]. Thus, using three different criteria for identifying apoptotic cell death, these data demonstrate that the saturated but not unsaturated FFA induces apoptosis in HMC, similar to the proapoptotic effects of saturated FFA in other cell types [5,6,8,11,13]. In addition, enhanced β-oxidation of palmitate is not involved in this process; indeed, increased disposal of palmitate via oxidation apparently protects mesangial cells.

### Palmitate activates an intrinsic proapoptotic signaling pathway in HMC

**Figure 3:**
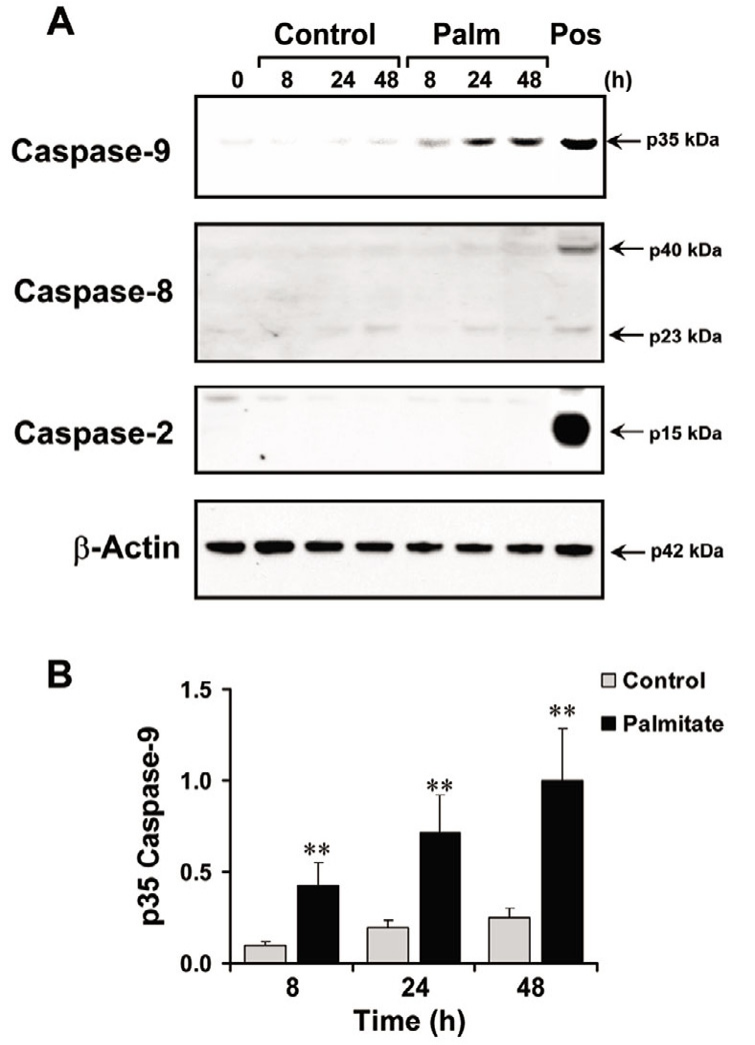
Palmitate stimulates cleavage of caspase 9 in HMC. (A) HMC treated with palmitate were analyzed for caspase cleavage by Western blotting of total HMC lysates with antibodies that recognize the cleavage products of human caspase 9 (p35), caspase-8 (p40 and p23), and caspase-2 (p15). For a positive control in each experiment, cells were treated for 8 h with an agent known to be a strong stimulus for the caspase in question: caspase 9, staurosporine 1 μM; caspase 8, etoposide 25 μM; and caspase 2, camptothecin 6 μM. The blots were reprobed with β-actin to ensure equal protein loading. (B) Densitometric analysis (mean ± SEM) of the p35 caspase 9 fragment from 3 independent experiments. **, *P *< 0.01 by ANOVA versus control.

We next investigated the signaling pathways by which palmitate induces apoptosis in HMC. To begin answering this question, we examined activation of initiator caspases associated with different pathways of apoptotic signaling. Cleavage of procaspase zymogens is required to form active heterotetrameric caspase complexes, so we analyzed the time course of caspase cleavage by Western blotting in 3 independent experiments. The p35 cleaved fragment of caspase 9 was elevated in cells treated with palmitate (Fig. 3A,B). After 48 h of palmitate, the amount of cleaved caspase 9 was similar to that in cells treated for 8 h with the robust apoptotic stimulus staurosporine (Fig. 3A, Pos lane). In contrast, palmitate did not increase cleavage of caspase-8 (Fig. 3A), but treatment with a strong caspase-8 activator, etoposide, confirmed that HMC expressed caspase-8 and that the Western blot correctly measured caspase-8 cleavage (Fig. 3A, Pos lane). Caspase-2 has recently been implicated as an initiator caspase in signals involving stress in the endoplasmic reticulum or nucleus [25]. However, palmitate did not induce caspase-2 cleavage in HMC whereas the known caspase-2 stimulus camptothecin activated robust cleavage of the p15 fragment (Fig. 3A). Reprobing of all blots for β-actin confirmed equal protein loading (Fig. 3A). Collectively, these results suggest that palmitate activates caspase 9, an important initiator caspase in the intrinsic pathway of proapoptotic signal transduction.

**Figure 4:**
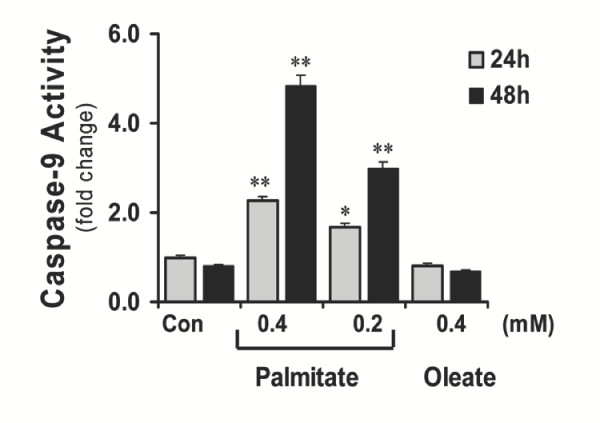
Palmitate but not oleate increases caspase 9 enzyme activity. HMC were treated with palmitate (0.2 and 0.4 mM) or oleate (0.4 mM) for 24 and 48 h. Caspase 9 activity was then measured in HMC extracts adjusted for total protein using a luminometric assay with an LEHD peptide substrate as described in Materials and Methods. Data are mean ± SEM for n = 3 experiments in duplicate. **, *P *< 0.01, *, *P *< 0.05 versus control or oleate alone by ANOVA.

To confirm that palmitate activated caspase 9, we used a luminometric caspase 9 substrate to directly measure caspase 9 enzyme activity in cytosolic extracts of HMC. Palmitate at 0.4 mM increased caspase 9 activity 2.2- and 4.8-fold at 24 and 48 h, respectively (Fig. 4). A lower dose of palmitate (0.2 mM) that also stimulated apoptosis (Fig. 2) increased caspase 9 activity. Importantly, oleate (0.4 mM) did not stimulate caspase 9 activity above control levels at any time point tested (Fig. 4). These results confirm that the saturated FFA palmitate increases caspase 9 enzyme activity.

**Figure 5:**
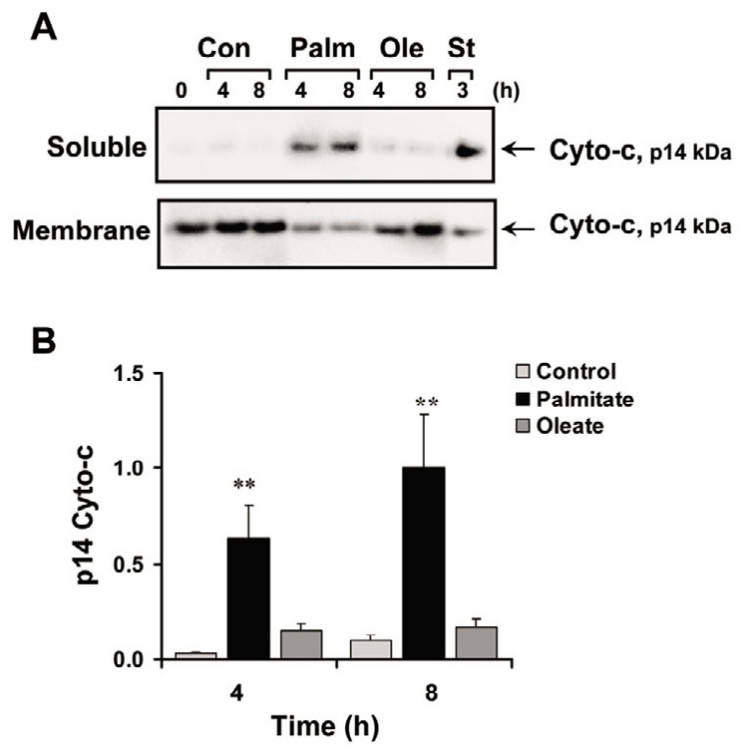
Palmitate induces translocation of cytochrome-c in cultured HMC. (A) Cells treated with control (Con, i.e., albumin alone), palmitate (Palm, 0.4 mM), oleate (Ole, 0.4 mM), or staurosporine (St, 1 μM) for the times indicated. HMC were then rapidly separated into soluble cytosolic and insoluble membrane fractions. The amount of cytochrome-c (Cyto-C) was determined by Western blotting. Equivalent amounts of total protein were present across all lanes of the cytoplasmic and membrane fractions, but the membrane fractions contained approximately 3 times more protein than the corresponding lane in the cytoplasmic fraction. (B) Densitometric analysis of cytoplasmic p14 kDa cytochrome-c from 3 independent experiments. Data are mean ± SEM. **, *P *< 0.01 by ANOVA versus control and oleate alone.

Because activation of caspase 9 by palmitate points to an intrinsic pathway of proapoptotic signaling, we asked whether palmitate could stimulate release of cytochrome-c from mitochondria. Redistribution of cytochrome-c to the cytoplasm is an important step in apoptosome formation and greatly enhances the enzymatic activity of caspase 9 [25,26]. Western blotting of cytoplasmic and membrane-enriched fractions that contain mitochondria was used to assess cytochrome-c distribution. In control cells (5% FBS plus albumin alone), cytochome-c resided exclusively in the membrane fraction (Fig. 5A,B). In cells treated with palmitate, a portion of total cytochrome-c was redistributed to the cytoplasmic fraction. Consistent with the inability of oleate to activate caspase 9, oleate did not appreciably redistribute cytochrome-c to the cytosol (Fig. 5). These results provide additional evidence that palmitate activates an intrinsic pathway of proapoptotic signaling in HMC.

### Inhibition of caspase 9 attenuates palmitate-induced apoptosis in HMC

**Figure 6:**
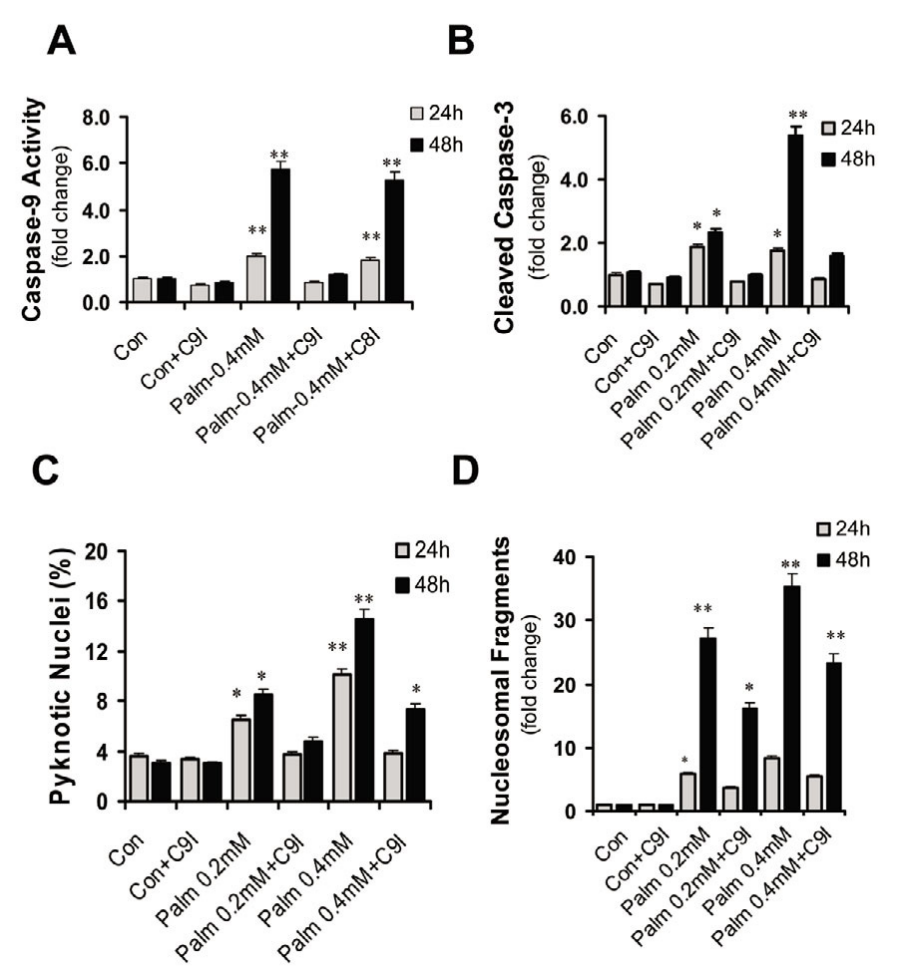
Effect of caspase 9 inhibition on apoptosis of HMC induced by palmitate. (A) Cells were treated with control (Con) or palmitate (Palm) in the presence and absence of the cell permeable caspase 9 inhibitor (C9I, Z-LEHD-FMK) or the caspase 8 inhibitor (C8I, Z-IETD-FMK), both at 40 μM. Addition of the caspase inhibitors was concurrent with addition of the FFA. After 24 and 48 h, caspase 9 enzyme activity was measured and expressed as fold-change over control. (B) Palmitate and Z-LEHD-FMK were added to HMC as above, and cleavage of caspase-3 was measured by ELISA as described in Experimental Procedures. (C) The number of pyknotic nuclei was assessed in cells treated with palmitate with and without Z-LEHD-FMK. (D) DNA fragmentation, assessed by ELISA as the number of nucleosomal fragments in the cytosol, was measured in cells treated with palmitate and by Z-LEHD-FMK. For A-D, data are mean ± SEM for 3 independent experiments. **, *P *< 0.01, *, *P *< 0.05 versus control by ANOVA in (A, B and D) or Chi-square test in (C).

We next tested the functional role of caspase 9 activation in the palmitate-induced signal transduction cascade. Co-incubation with a cell-permeable selective inhibitor of caspase 9 blocked activation of caspase 9 enzyme activity by palmitate (Fig. 6A). The caspase 9 inhibitor alone had a minor effect (<0.2-fold inhibition) on basal caspase 9 activity, and a caspase-8 inhibitor did not block caspase 9 activity stimulated by palmitate (Fig. 6A). Under theses conditions, inhibition of caspase 9 abolished caspase-3 cleavage in cells treated with palmitate for 24 and 48 h (Fig. 6B). Formation of pyknotic nuclei by 0.2 mM palmitate was blocked in cells treated with the caspase 9 inhibitor at both 24 and 48 h. However, the number of pyknotic nuclei was only partially reduced in cells treated with 0.4 mM palmitate for 48 h (Fig 6C), even though under these conditions caspase 9 enzyme activity was completely inhibited (Fig. 6A). Similarly, DNA fragmentation by palmitate was incompletely blocked by the caspase 9 inhibitor at 48 h (Fig. 6D). These results show that inhibition of palmitate-stimulated caspase 9 blocks some but not all apoptotic death in HMC. Because the caspase 9 inhibitor completely blocked caspase-3 cleavage, these results also suggest that proapoptotic signaling by palmitate proceeds by both caspase 9/3-dependent and independent mechanisms.

### Mitochondrial release of endonuclease G in palmitate-treated HMC

**Figure 7:**
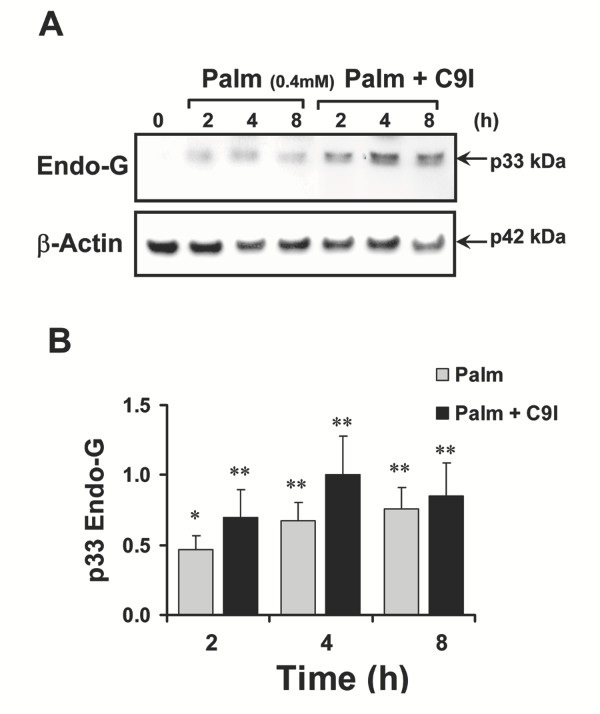
Palmitate stimulates translocation of the proapoptotic endonuclease G protein by a caspase 9-independent mechanism. (A) HMC were incubated with palmitate (Palm) and palmitate plus the Z-LEHD-FMK caspase 9 inhibitor (C9I, 40 μM) for the times indicated. The cells were fractionated and the level of p33 endonuclease G protein was measured by Western blotting in the cytoplasmic fraction. Equivalent amounts of protein were added to each lane, as confirmed by reprobing with β-actin. (B) Densitometry of cytoplasmic p33 kDa endonuclease G protein from 3 independent experiments. **, *P *< 0.01, *, *P *< 0.05 by ANOVA versus 0 time.

To elucidate the caspase 9/3-independent mechanisms of proapoptotic signaling by palmitate, we investigated the possibility that palmitate stimulates the release of other mitochondrial proapoptotic proteins that can contribute to nuclear changes independent of casapse-3. Endonuclease G is one such effector of apoptosis, a mitochondrial DNase released by a Bcl-2-dependent but caspase-independent mechanism [27]. To examine the extent to which palmitate can induce endonuclease G release, we treated HMC with palmitate and measured the presence of endonuclease G in the cytoplasm of fractionated cells. Palmitate increased the amount of endonuclease G released into the cytoplasm (Fig. 7A and 7B). The release of endonuclease G in palmitate-treated cells was not altered by caspase 9 inhibition (Fig. 7A and 7B). These results are consistent with the incomplete inhibition of DNA fragmentation observed when caspase 9 activation by palmitate was blocked in HMC (Fig. 6D). Collectively, these results suggest that palmitate-induced apoptosis proceeds by caspase 9-dependent mechanisms and by a caspase 9 -independent mechanism involving endonuclease G.

### Caspase 9-dependent and -independent pathways of palmitate-induced apoptosis are abolished by unsaturated FFA

**Figure 8:**
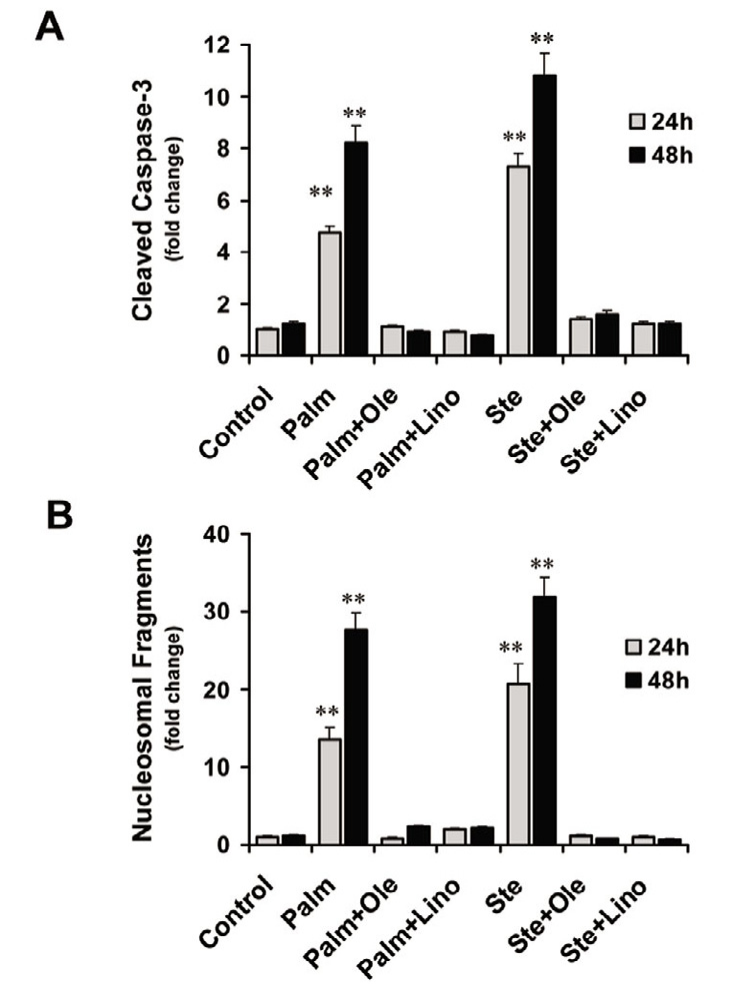
Unsaturated FFA block apoptosis induced by saturated FFA. HMC were incubated for 24 or 48 h with 0.2 mM palmitate (Palm) or stearate (Ste) alone or in co-incubations with 0.2 mM of the unsaturated FFA oleate (Ole) or linoleate (Lin). Control cells were incubated with albumin alone. After 24 and 48 h, the level of (A) the cleaved fragment of caspase-3 and (B) cytoplasmic nucleosomal fragments were determined by ELISA and expressed relative to the 24 h control value. Data are mean ± SEM for n = 3 independent experiments in triplicate. **, *P *< 0.01 by ANOVA versus control.

**Figure 9:**
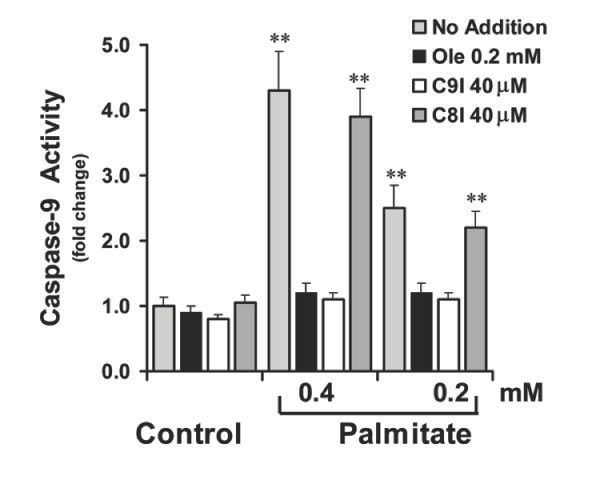
Oleate inhibits caspase 9 activation by palmitate. HMC were treated with palmitate (0.2 and 0.4 mM) or palmitate plus oleate (0.2 mM) for 24 and 48 h. Caspase 9 activity was then measured in HMC extracts adjusted for total protein. In some experiments the cells were treated with palmitate plus selective inhibitors of caspase 9 (C9I) or caspase 8 (C8I) at 40 μM. Data are mean ± SEM for n = 3 experiments in duplicate. **, *P *< 0.01, *, *P *< 0.05 versus control or palmitate plus oleate by ANOVA.

Unsaturated FFA have been reported to block apoptosis by saturated FFA [8,11,13], so we next asked whether unsaturated FFA block palmitate-induced apoptosis in HMC and whether they act by inhibiting the caspase 9-dependent or -independent pathway. The unsaturated FFA oleate and linoleate blocked the increase in caspase-3 cleavage and DNA fragmentation in cells treated with palmitate (Fig. 8A,B). Apoptosis induced by stearate was also effectively blocked in cells co-incubated with oleate or linoleate (Fig. 8A,B). Oleate also prevented the increase in caspase 9 activity in cells treated with palmitate (Fig. 9). The specificity of this assay for caspase 9 was confirmed by showing that the cell-permeable caspase 9 inhibitor prevented palmitate-stimulated enzyme activity whereas a caspase 8-selective inhibitor had no effect (Fig. 9). Thus, similar to other cell types, unsaturated FFA inhibit the apoptotic response to saturated FFA in HMC. Moreover, oleate and linoleate completely prevent palmitate-induced apoptosis, which contrasts with the partial blockade observed with inhibition of caspase 9 (Fig. 6).

**Figure 10:**
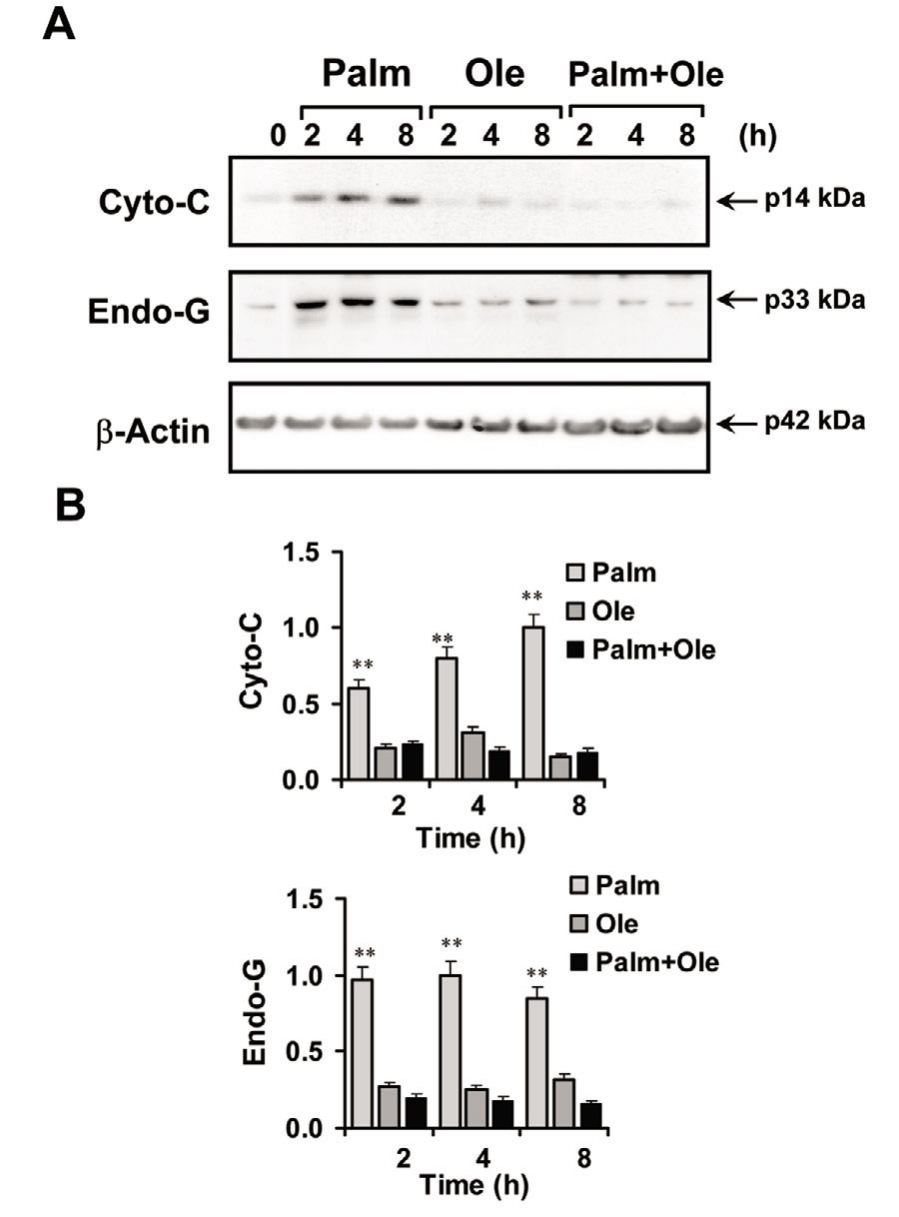
Oleate prevents mitochondrial release of cytochrome-c and endonuclease G in cell treated with palmitate. (A) HMC were incubated with 0.2 mM palmitate (Palm), 0.2 mM oleate (Ole), or palmitate plus oleate for the times indicated. The cells were fractionated and the levels of cytoplasmic p14 kDa cytochrome-c (Cyto-c) or p33 kDa endonuclease G (Endo-G) protein were measured by Western blotting. Equivalent amounts of protein were added to each lane, as confirmed by reprobing with β-actin. (B) Densitometry of cytoplasmic p14 kDa cytochrome-c and p33 kDa endonuclease G protein from 3 independent experiments. **, *P *< 0.01, *, *P *< 0.05 by ANOVA versus 0 time.

Because the unsaturated FFA more effectively blocked palmitate-induced apoptosis, we investigated whether this was because they blocked the caspase-independent release of endonuclease G in cells treated with palmitate. As expected, palmitate increased mitochondrial release of cytochrome-c and endonuclease G in HMC (Fig. 10A,B). Oleate alone had no effect on release of cytochrome-c or endonuclease G. Co-incubation of oleate with palmitate prevented the release of cytochrome-c and endonuclease G induced by palmitate (Fig. 10A,B). These results are consistent with the notion that oleate blocks both the caspase 9-dependent and -independent mechanisms of proapoptotic signaling by palmitate. In particular, the ability of oleate to block release of endonuclease G in palmitate-treated cells is one explanation for the superior antiapoptotic effect of oleate compared to caspase 9 antagonism.

## Discussion

Our results show that the saturated FFA palmitate induces an intrinsic pathway of proapoptotic signaling in HMC, a vascular target cell of lipid-mediated injury in the kidney. In contrast, the monounsaturated FFA oleate did not induce proapoptotic signaling and instead protected HMC from palmitate-induced apoptosis. Evidence that palmitate induced an intrinsic pathway of proapoptotic signaling is that it increased cytochrome-c release, caspase 9 cleavage, and caspase 9 enzyme activity. We also showed that the palmitate-stimulated intrinsic pathway proceeded by a caspase 9-dependent mechanism and by a caspase 9 -independent mechanism involving endonuclease G. Both the caspase 9-dependent and -independent pathways were effectively blocked by oleate.

In this study we report that palmitate causes apoptosis in HMC, a microvascular cell that accumulates lipids in vivo in settings of high FFA such as obesity and type 2 diabetes [15-20]. We chose palmitate because it is the most abundant saturated FFA complexed to human serum albumin [28]. We used complexes where the molar ratio of FFA to albumin was 6:1. Although the normal physiologic ratio of FFA to albumin is approximately 2:1, serum FFA levels are greatly elevated in patients with obesity, type 2 diabetes, and proteinuric renal diseases, yielding ratios of 6:1 or higher [29,30]. In addition, normal circulating FFA levels are approximately 0.5 mM [3], so the concentrations of individual FFA species (i.e., 0.2 – 0.4 mM) used in this study seem reasonable. Therefore, our experiments were designed to evaluate mechanisms of FFA-induced apoptosis relevant to type 2 diabetes.

Previous studies have demonstrated that saturated but not monounsaturated FFA cause apoptosis in other non-renal cell types [5,6,8,11,13], but the ability of FFA to induce apoptosis appears to vary with the specific cell type in question [1]. In addition, the signal transduction pathways by which saturated FFA induce apoptosis are incompletely defined. Similar to a previous report in breast cancer cells [13], palmitate-induced apoptosis in HMC was enhanced by inhibition of fat oxidation and reversed by increasing fat oxidation. Although we did not directly measure the effects of these compounds on fatty acid oxidation, these results suggest that palmitate must be metabolized to promote apoptosis and that mitochondrial β-oxidation of the saturated FFA does not participate in the proapoptotic response to palmitate. Several observations from our study suggest that palmitate induces an intrinsic pathway of apoptotic signaling in HMC. First, palmitate stimulated accumulation of cytochrome-c in the cytosol, which is an important step in the intrinsic pathway to promote apoptosome formation and activation of caspase 9. Palmitate-induced release of cytochrome-c has been previously reported in β-cells and breast cancer cells [13,14]. Palmitate has also been shown to stimulate release of uncharacterized proapoptotic proteins when added directly to isolated mitochondria [31]. Second, palmitate stimulated caspase 9 cleavage and activity in HMC. To our knowledge activation of caspase 9 by palmitate has not been previously shown. Caspase 9 is an initiator caspase in many but not all intrinsic pathways of proapoptotic signaling [25,26]. In a stimulus- and cell type-specific manner, caspase 2 can function upstream of caspase 9 [32-35], but in our experiments palmitate did not stimulate cleavage of caspase 2, an indicator of caspase 2 activation. Also, we did not observe cleavage of caspase 8, an initiator caspase in the death receptor pathway, in response to palmitate. Thus an important result of our study is that the intrinsic pathway in palmitate-induced apoptosis appears to involve caspase 9.

A functional role for caspase 9 in palmitate-induced apoptosis was suggested by experiments in which a cell-permeable caspase 9 inhibitor, Z-LEHD-FMK, blocked apoptosis in response to palmitate. Z-LEHD-FMK completely inhibited the activation of caspase 9 at 24 and 48 h of 0.4 mM palmitate. Under these conditions, Z-LEHD-FMK reversed caspase-3 cleavage induced by palmitate, suggesting that caspase 9 is required for activation of caspase-3 by palmitate. While Z-LEHD-FMK effectively inhibited the nuclear changes characteristic of apoptosis at 24 h, the caspase 9 inhibitor did not completely reverse pyknotic nuclei or DNA fragmentation at 48 h. For example, partial inhibition of DNA fragmentation was observed in HMC treated with 0.2 or 0.4 mM palmitate for 48 h, even though the palmitate-induced increment in caspase 9 was blocked. A possible explanation of these results is that when HMC are exposed to palmitate for 48 h, the saturated FFA recruits additional caspase 9-independent mechanisms of apoptotic or non-apoptotic cell death that are not induced at 24 h. A possible caspase 9-independent mechanism for palmitate-induced apoptosis would be the mitochondrial release of endonuclease G, which we demonstrated in HMC. Endonuclease G is a DNase normally located in the intermembrane space of mitochondria. Some apoptotic stimuli cause caspase-independent release of endonuclease G after which it translocates to the nucleus and cleaves DNA [27]. Release of endonuclease G in palmitate-treated HMC was not blocked by Z-LEHD-FMK, which could explain the partial inhibition of DNA fragmentation by Z-LEHD-FMK under conditions where caspase 9 activation by palmitate was completely blocked. It is also possible that a small amount of caspase 3 activity remains even in the presence of the caspase 9 inhibitor, and it is possible that this low level of caspase-3 activity also contributes to endonuclease G release.

In striking contrast to the partial inhibition of palmitate-induced apoptosis by the caspase 9 antagonist, we found that the unsaturated FFA oleate and linoleate completely prevented caspase 3 cleavage and DNA fragmentation in cells treated with either palmitate or stearate. Oleate prevented mitochondrial release of cytochrome-c and the increase in caspase 9 in cells treated with palmitate. In addition, oleate blocked mitochondrial release of endonuclease G in palmitate-treated cells. Taken together, these results support the notion that oleate completely prevents palmitate-induced apoptosis because, unlike inhibition of caspase 9 alone, oleate blocks both the caspase 9-dependent and -independent pathways.

## Conclusions

These results show that palmitate stimulates apoptosis by evoking an intrinsic pathway of proapoptotic signaling. In addition, we have identified mitochondrial release of endonuclease G as a key step in proapoptotic signaling by saturated FFA and in the anti-apoptotic actions of unsaturated FFA. We believe that these results might be relevant to the pathogenesis of microvascular injury in type 2 diabetes because FFA accumulate in microvascular cells, including mesangial cells, *in vivo *in experimental models of type 2 diabetes, obesity, or hyperlipidemia [15-20]. Thus lipid-driven apoptosis might contribute to the microvascular remodeling that leads to numerous complications in type 2 diabetes.

## List of Abbreviations

FFA, free fatty acid; HMC, human mesangial cells;

## Competing Interests

The author(s) declare that they have no competing interests.

## Authors' contributions

RM carried out most of the technical studies and helped draft the manuscript. MSS conceived of the study, participated in its design and execution, and helped to draft the manuscript.
